# Acute polymicrobial airway infections: analysis in cystic fibrosis mice

**DOI:** 10.1099/mic.0.001290

**Published:** 2023-01-25

**Authors:** Natalie R. Lindgren, Melissa S. McDaniel, Lea Novak, W. Edward Swords

**Affiliations:** ^1^​ Department of Medicine, Division of Pulmonary, Allergy, and Critical Care Medicine, University of Alabama, Birmingham, USA; ^2^​ Gregory Fleming James Center for Cystic Fibrosis Research, University of Alabama, Birmingham, Birmingham, USA; ^3^​ Department of Microbiology, University of Alabama at Birmingham, Birmingham, USA

**Keywords:** airway infection, bacteria, Cystic fibrosis, polymicrobial

## Abstract

Cystic fibrosis (CF) is a genetic disorder affecting epithelial ion transport, which among other impacts results in defective mucociliary clearance and innate defenses in the respiratory tract. Consequently, people with CF experience lifelong infections of the respiratory mucosa that are chronic and polymicrobial in nature. Young children with CF are initially colonized by opportunists like nontypeable *Haemophilus influenzae* (NTHi), which normally resides within the microbiome of the nasopharynx and upper airways and can also cause infections of the respiratory mucosa that include bronchitis and otitis media. NTHi is typically supplanted by other microbes as patients age; for example, people with CF are often chronically infected with mucoid strains of *Pseudomonas aeruginosa *, which prior work in our laboratory has shown to promote colonization and persistence by other opportunists that include *Stenotrophomonas maltophilia *. Our previous work has shown that polymicrobial infection impacts host colonization and persistence of incoming microbes via diverse mechanisms that include priming of host immunity that can promote microbial clearance, and cooperativity within polymicrobial biofilms, which can promote persistence. In infection studies with BALB/c Cftr^tm1UNC^ mice, results showed, as previously observed for WT BALB/c mice, preceding infection with NTHi decreased colonization and persistence by *P. aeruginosa *. Likewise, polymicrobial infection of BALB/c Cftr^tm1UNC^ and C57BL/6 Cftr^tm1UncTg^(FABPhCFTR)1Jaw/J mice showed correlation between *S. maltophilia* and *P. aeruginosa *, with increased bacterial colonization and lung pathology. Based on these results, we conclude that our previous observations regarding polymicrobial infections with CF opportunists in WT mice are also validated using CF mice.

## Introduction

Cystic fibrosis (CF) is a genetic disorder caused by mutations affecting an epithelial ion transporter, the cystic fibrosis transmembrane conductance regulator (CFTR) [1, 2]. CFTR deficits have many pathological consequences, including impairment of mucociliary clearance and innate immune defenses in the airways. As a result, people with CF experience lifelong opportunistic infections of the airway mucosa, which are significant contributors to CF morbidity and mortality [3, 4]. The microbiome of the lungs of persons with CF are complex, dynamic and constantly changing, and undergoes predictable shifts in dominant microbial phyla that can correlate with changes in respiratory function and overall health [5–7]. Defining mechanisms that govern interbacterial cooperation and/or competition within the CF lung, and their impacts on patient health outcomes are central questions in our work on CF-related infections.

Children experience colonization of the nasopharynx and upper airways early in life with airway commensals such as nontypeable *Haemophilus influenzae *, *Moraxella catarrhalis* and *Streptococcus pneumoniae *; for children with CF, the carriage of these organisms extends throughout the lung and lower airways [8, 9]. As patients age, these microbes are gradually supplanted by other bacterial species, most notably mucoid variants of *Pseudomonas aeruginosa,* which often correlates with declining respiratory function and worsened disease outcomes [10, 11]. Our recent work showed that in experimental mouse respiratory infections, NTHi diminishes colonization and persistence of *P. aeruginosa* by priming of innate host defenses [12]. Conversely, in separate studies, we also showed that *P. aeruginosa* can promote colonization of other bacteria, namely *Stenotrophomonas maltophilia *, by means of cooperative persistence within polymicrobial biofilms [13]. In this work, we extend these findings to the context of CF using CF mutant mice. Mice harbouring CFTR^tmc1UNC^ mutations were the first CF animal models generated for research use; however, inherent intestinal obstruction notoriously led to the premature demise of these mice; in response, bitransgenic mice expressing rat fatty acid binding protein 2 and an intestinal promotor for human CFTR (FABPhCFTR) were generated, extending survival to maturity [14]. Herein, CF mutant mice with CFTR^tmc1UNC^ mutations in two genetic backgrounds [BALB/c CFTR^tmc1UNC^ or C57Bl/6 Cftr^tm1UncTg^(FABPhCFTR)1Jaw/J] were infected with various combinations of bacterial species as in the previous studies, and compared at differing time points post-infection for lung bacterial burden, immune cell influx into bronchoalveolar lavage fluid (BALF), histopathological damage and cytokine/chemokine markers of immunological response. Our findings suggest that previously established polymicrobial interactions do not differ in these CF models compared to non-CF mice, but that the background of these CF mouse models can impact immune sequelae and pathology following infection.

## Methods

### Bacterial strains and growth conditions

For *S. maltophilia* and *P. aeruginosa* studies, strain *S. maltophilia* K279a, a widely used model strain with a fully annotated genome, was provided by M. Herman (Kansas State University) [15]. Strain *P. aeruginosa* mPA08-31, a sputum-derived CF clinical isolate, was obtained from S. Birket (University of Alabama at Birmingham). All strains were routinely cultured on Luria–Bertani (LB) agar (Difco) or in LB broth. *S. maltophilia* was streaked for colony isolation before inoculation into LB broth and shaking overnight at 30 °C and 200 r.p.m. *P. aeruginosa* was streaked for colony isolation before inoculation into LB broth and shaking overnight at 37 °C and 200 r.p.m.

For nontypeable *H. influenzae* and *P. aeruginosa* studies, strain NTHi HI-1, a CF clinical isolate, was provided by Timothy Starner, University of Iowa Children’s Hospital. NTHi was routinely cultured on supplemented brain-heart infusion (sBHI) agar (RPI, ThermoFisher Scientific), containing 10 µg ml^−1^ of hemin (Sigma Aldrich, St. Louis, MO, USA) and 1 µg ml^−1^ of NAD (Sigma Aldrich). NTHi was cultured on supplemented BHI agar, incubated overnight at 37 °C+5 % CO_2_. Strain *P. aeruginosa* mPA 08–31 was cultured in LB broth and shaking overnight at 37 °C and 200 r.p.m.

### Mouse model of infection

Cystic fibrosis mouse models, including BALB/c Cftr^tm1UNC^ (JAX no. 002196) and C57BL/6J- Cftr^tm1UncTg^(FABPhCFTR)1Jaw/J (JAX no. 002364) backgrounds with CFTR null mutations, were obtained from the Cystic Fibrosis Mouse Model core at the University of Alabama at Birmingham (UAB). All mice received supplemental diet, in addition to standard chow, of DietGel dietary supplement (ClearH2O, Westbrook, ME, USA) and Pedialyte (Abbott, Chicago, IL, USA). At 6–8 weeks of age, mice were anesthetized with isoflurane and intratracheally infected via tracheal cannulation. In short, anesthetized mice were vertically suspended on an intubation board (BioLite, Braintree Scientific, Braintree, MA, USA) blunt forceps were used to position the tongue, and bacterial inoculum was administered directly into the trachea via a 21 gauge cannula attached to a syringe. Mice were allowed to hang vertically until all liquid was aspirated and were closely monitored until respiration returned to normal. Uninfected control mice were administered a volume equivalent dose of PBS vehicle (100 µl). Weight was recorded prior to infection and monitored every 24 h post-infection. Weight loss was not reported for animals that expired prematurely. All mouse infection protocols were approved by the UAB Institutional Animal Care and Use Committees.

For nontypeable *H. influenzae* and *P. aeruginosa* studies, mice were infected with ~10^7^ c.f.u. ml^−1^ of NTHi followed 24 h later by ~10^8^ c.f.u. ml^−1^ of *P. aeruginosa *, essentially as we have described previously [12]. Endpoints of respiratory infection were assessed 72 h post-dual challenge. To determine the bacterial burden of the lungs, the left lung of each mouse was harvested and homogenized with stainless steel beads in 500 µl of sterile PBS (30 Hz s^−1^ for 3 min). Lung homogenates were serially diluted in PBS and plated on selective media. NTHi bacteria were enumerated by plate count using sBHI agar, except for coinfected mice in which case the NTHi were enumerated on sBHI with spectinomycin (2 mg ml^−1^) for selection of NTHi. Animals that expired before the end of the study were shown in bacterial enumeration counts as 1×10^9^ c.f.u./lung.

For *S. maltophilia* and *P. aeruginosa* studies, mice were infected with ~10^8^ c.f.u. ml^−1^ of each species and endpoints were assessed 24 h post-infection. Lung homogenates were serially diluted in PBS and plated on selective media. Single-species infected lungs were plated on LB, while dual-species infected lungs were plates on LB agar containing gentamicin (50 µg ml^−1^) for quantification of *S. maltophilia *. Animals that expired before the end of the study were shown in bacterial enumeration counts as 1×10^9^ c.f.u./lung.

### Histological analyses

For histological analyses, the right lung of each mouse was perfused with PBS, inflated with 10% buffered formalin (ThermoFisher Scientific, Waltham, MA, USA), and stored at 4 °C until processing. Slices from each lobe of the right lung were trimmed and sent to the UAB Comparative Pathology Laboratory to be processed, paraffin embedded, sectioned onto slides and stained with hematoxylin and eosin (H and E). Semiquantitative grading of histopathological changes of all lung sections was performed by a board-certified surgical pathologist (L.N.). Severity of lung tissue injury included alveolar, peribronchial inflammation, perivascular neutrophil infiltration, pleuritis and tissue necrosis. Severity of tissue damage from all lung lobes was graded. Grade 0 showed no neutrophil infiltrate in lung tissue; grade 1 showed rare neutrophils in the alveolar, peribronchial or perivascular tissue; grade 2 showed dense neutrophil infiltrate within the alveolar spaces with no injury to alveolar tissue; grade 3 showed dense neutrophil infiltrate within the alveolar space and necrosis of involved alveolar tissue. H-score is a cumulative score, determined by the percentage of tissue area assigned each grade (0–3). Maximum grade was assigned to the most severely damaged lobe, representing the maximum infection per animal. Images of lung sections were taken on a Lionheart FX Automated Imaging microscope (BioTek, Winooski, VT, USA) using 10× objective. Scale bar represents 200 µm.

### Bronchoalveolar lavage fluid

Bronchoalveolar lavage was performed by flushing lungs with 5–6 ml of PBS in 1 ml increments as previously described [12, 16]. Collected bronchoalveolar lavage fluid (BALF) was stored on ice until processing. The first 1 ml increment was centrifuged (1350 ***g***, 5 min) to separate the supernatant from immune cells and the cell pellet was used for total and differential cell counts. The remaining 4–5 ml of collected BALF were centrifuged to remove debris (1350 ***g***, 5 min) and supernatant was stored at −20 °C until use for cytokine analyses.

### Differential cell counting and cytokine analyses

For total and differential cell counts, cell pellets from BALF were resuspended in sterile PBS and mounted on a slide by cytospin (500 r.p.m. for 5 min). Cells were stained with a Kwik-Diff differential cell stain (ThermoFisher). Three representative fields of view from each slide were counted to determine immune cell counts. Cytokine/chemokines were measured in undiluted BALF by a custom Milliplex mouse Cytokine magnetic panel (MilliporeSigma, St. Louis, MO, USA).

### Statistical analyses

Graphs depict sample means±standard error of the mean (sem). Mouse numbers within each group are denoted in the figure legends. Following the Shapiro–Wilk normality test, nonparametric data sets were analysed by the Kruskal–Wallis test with the uncorrected Dunn’s test for multiple comparisons. Comparisons between CFTR mutant mice and their WT littermates were evaluated against infection status (two factors), measured by two-way ANOVA with Sidak’s multiple comparison test; *P*-values≤0.05 were considered statistically significant. All statistical tests were performed using GraphPad Prism 9 (San Diego, CA, USA).

## Results

### Preceding infection with nontypeable *H. influenzae* decreases colonization/persistence of *P. aeruginosa* in BALB/c Cftr^tm1UNC^ mice

**Fig. 1.**
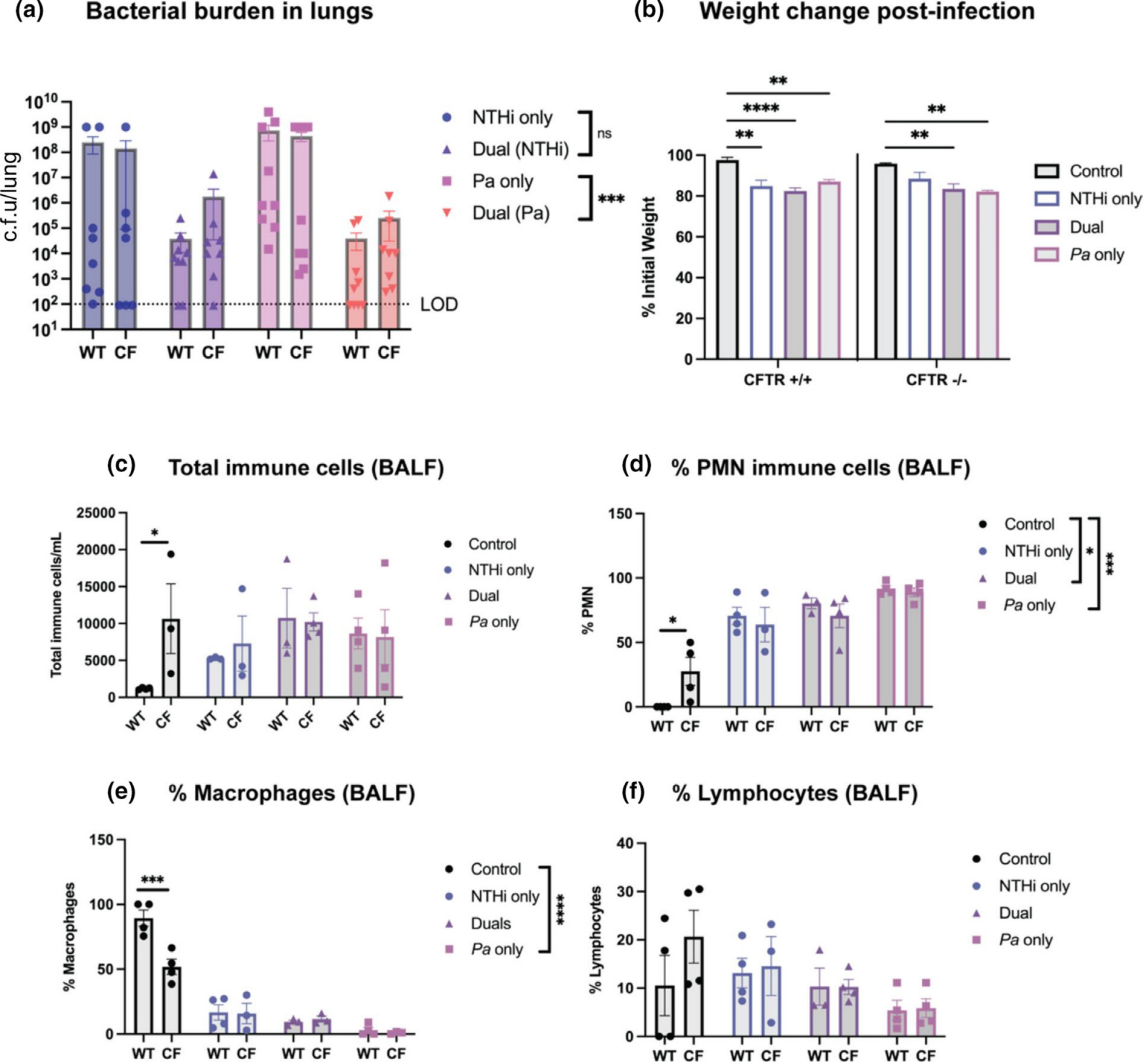
Nontypeable *Haemophilus influenzae* and *Pseudomonas aeruginosa* dual-species respiratory infections in BALB/c mice. BALB/c Cftr^tm1UNC^ mice and WT littermates were intratracheally infected with ~10^8^ c.f.u. ml^−1^ of nontypeable *H. influenzae *, followed by ~10^7^ c.f.u. ml^−1^ of *P. aeruginosa* 24 h later. (a) Bacterial burden in the lung homogenate was enumerated by viable colony counting after 48 h of dual infection. LOD=limit of detection. (*N*=7–9). (b) Weight loss was measured as percent initial weight at 48 h post-infection. (*N*=4–9). Two-way ANOVA with Sidak’s multiple comparison test. Mean±sem, **P<0.01, ****P<0.0001. BALF was collected for differential cell counts of immune cells, including (c) total immune cells, (d) the percentage of total cells that are PMN, (e) the percentage of total cells that are macrophages, and (f) the percentage of total cells that are lymphocytes (*N*=4). Comparisons between CF mice and their WT littermates were evaluated by two-way ANOVA with Sidak’s multiple comparison test. Mean±sem, *P<0.05, ***P<0.001. Comparisons between bacterial infection groups were evaluated by Kruskal–Wallis with Dunn’s multiple comparison test. Mean±sem, *P<0.05, **P<0.01.

In previous work, we showed that preceding infection with nontypeable *H. influenzae* impacted subsequent infection with *P. aeruginosa *, resulting in decreased bacterial burden and airway tissue damage documented by histopathologic examination [12]. To study these CF pathogens in a disease-specific model, we compared infection outcomes of BALB/c Cftr^tm1UNC^ mice to that of WT, age matched littermates. Mice were intratracheally infected with NTHi HI-1 followed 24 h later by *P. aeruginosa* (Fig S1A, available in the online version of this article). As expected, at 48 h post-dual infection, the bacterial burden of *P. aeruginosa* recovered from lung homogenate was significantly reduced in mice with prior NTHi challenge compared to mice infected with *P. aeruginosa* alone (***P<0.001). Interestingly, there were no statistically significant differences in bacterial counts between WT and CF mice within each group (Fig. 1a). To evaluate overall animal health, percent initial weight was calculated as a metric of weight loss over the course of infection. In both WT and CF mice from each infection group, bacterial challenge resulted in significant weight loss compared to mock-infected controls receiving vehicle (PBS), with no difference between WT and CF groups (Fig. 1b). Immune cell infiltrates in BALF were quantified by cytospin preparation and differential staining. There were no significant differences noted in total immune cell infiltrates between bacterial infection groups, and a difference in total immune cell counts between WT and CF groups only under mock-infected conditions (***P<0.001) (Fig. 1c). The percentage of PMN in BALF following *P. aeruginosa* single-species infection were elevated as compared to other groups, and were significantly increased compared to uninfected controls (***P<0.001). However, there was no significant difference in PMN percentage between WT and CF groups, except for between uninfected CF mice and their WT littermates (*P<0.05) (Fig. 1d). The composition of total cells made up of macrophages and lymphocytes was inversely proportional to that of PMN percentage, with *P. aeruginosa* infection resulting in a lesser percentage of macrophages compared to uninfected controls (****P<0.0001), and there was no difference in macrophage percentage or lymphocyte percentage between WT and CF groups, with an exception being macrophage percentage between uninfected controls (***P<0.001) (Fig. 1e-f). Additionally, animal mortality was markedly increased by *P. aeruginosa* single-species infection, with 11.1 % mortality in WT and 44.4 % mortality in CF mice (Table S1). Overall, these findings support prior work in which preceding infection with NTHi reduces bacterial colonization/persistence of *P. aeruginosa *. This is particularly notable given the significant upper airway microbiome we have observed in most CF mutant mice. In our experience the mice have chronic nasopharyngeal colonization with a range of microbial species (data not shown), which could conceivably provide innate priming in the absence of any artificial infection. Based on these results we conclude that preceding infection with NTHi invokes innate immune priming that can significantly impact subsequent infections.

**Fig. 2.**
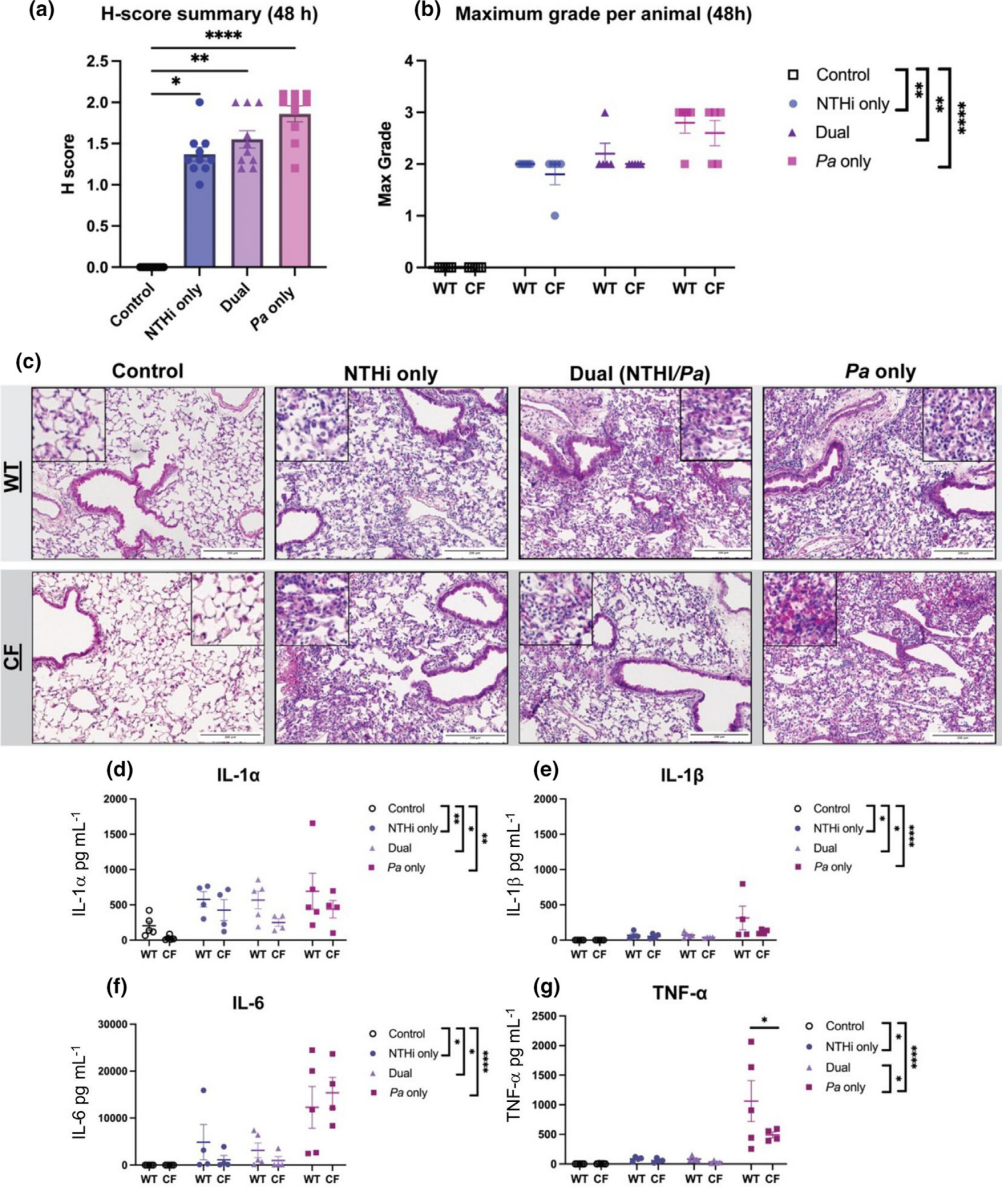
Histopathological analysis of BALB/c Cftr^tm1UNC^ and respiratory inflammatory responses in mice following infection with nontypeable *Haemophilus influenzae* and *Pseudomonas aeruginosa *. Lung sections from BALB/c Cftr^tm1UNC^ mice and WT littermates were evaluated and graded for tissue damage (scale 0–3) following nontypeable *H. influenzae* and *P. aeruginosa* dual-species infection. (a) H-score reflects a cumulative tissue grade (*N*=10). Kruskal–Wallis with Dunn’s multiple comparison test. Mean±sem, *P<0.05, **P<0.01, ****P<0.0001. (b) Maximum grade was assigned to the most severely damaged lobe (*N*=5). Comparisons between CFTR mutant mice and their WT littermates were evaluated by two-way ANOVA with Sidak’s multiple comparison test. Statistical analyses were performed using Kruskal–Wallis with Dunn’s multiple comparison test. Mean±sem, **P<0.01, ****P<0.0001. Pathological grading by L.N. Representative images of (c) H and E-stained lung sections comparing CF mice and their WT littermates after bacterial infection. Rare macrophage presence in the alveoli of uninfected CF mice is not indicative of infection. Scale bar represents 200 µm. Cytokine/chemokine analyses of BALF supernatant from infected or mock-infected BALB/c Cftr^tm1UNC^ mice and WT littermates following nontypeable *H. influenzae* and *P. aeruginosa* infection. Luminex multiplex assay measured (d) IL-1α, (e) IL-1β, (f) IL-6 and (g) TNF-α (*N*=4–5). Comparisons between CF mice and their WT littermates were evaluated by two-way ANOVA with Sidak’s multiple comparison test. Mean±sem, *P<0.05. Statistical analyses were performed using Kruskal–Wallis with Dunn’s multiple comparison test. Mean±sem, *P<0.05, **P<0.01, ****P<0.0001.

Hematoxylin and eosin (H and E) stained lung sections from BALB/c Cftr^tm1UNC^ mice were microscopically evaluated for lung tissue damage by a pathologist (L.N.). The percentage of tissue areas categorized as specific grades (0–3) were calculated as a cumulative H-score. Following NTHi and *P. aeruginosa* infection, each infection group resulted in significant histopathological changes noted in the mice infected with *P. aeruginosa* alone (****P<0.0001) (Fig. 2a). The maximum grade following infection, representing the highest grade per animal, shows similar findings, with single-species *P. aeruginosa* infection resulting in the most severe damage compared to uninfected controls (****P<0.0001). However, no significant difference in score was found between WT and CF animal groups (Fig. 2b). Representative images of these lung sections indicate the severity of lung tissue injury following *P. aeruginosa* infection showing neutrophil influx and airway remodelling (Fig. 2c). Herein, we note that prior infection with NTHi reduces airway tissue damage caused by *P. aeruginosa *. These findings also indicate that lung pathology of the BALB/c Cftr^tm1UNC^ mice does not significantly differ from WT mice in regard to lung tissue damage at this timepoint.

Our previous work showed that sequential introduction of NTHi followed by *P. aeruginosa* resulted in significant reduction of some inflammatory markers, including MCP-1, TNF-α and anti-inflammatory marker IL-10 in dual-infected mice compared to a single-species *P. aeruginosa* infection. Other inflammatory makers, including IL-1α, IL-1β and IL-6, were not different between infection groups [12]. To assess this phenotype in the BALB/c Cftr^tm1UNC^ mouse model, we measured inflammatory markers in the BALF by multiplex assay. Infection with *P. aeruginosa* resulted in increased IL-1α compared to uninfected controls, with dual-species infection resulting in a slight decrease in this marker compared to single-species infections (*P<0.05) (Fig. 2d). Markers IL-1β, IL-6 and TNF-α also decreased following polymicrobial infection as compared to single-species *P. aeruginosa* infection, which was significantly higher than uninfected controls (****P<0.0001) (Fig. 2e-g). When comparing WT mice to their CF littermates, all measurements indicate no difference between groups, with an exception being that TNF-α levels were slightly higher in WT mice compared to CF mice (*P<0.05) (Fig. 2g). Overall, NTHi and *P. aeruginosa* infections in BALB/c Cftr^tm1UNC^ mice result in similar inflammatory cytokine levels to previous findings in BALB/c mice at this timepoint.

### *S. maltophilia* and *P. aeruginosa* colonization and correlation in BALB/c Cftr^tm1UNC^ mice

**Fig. 3.**
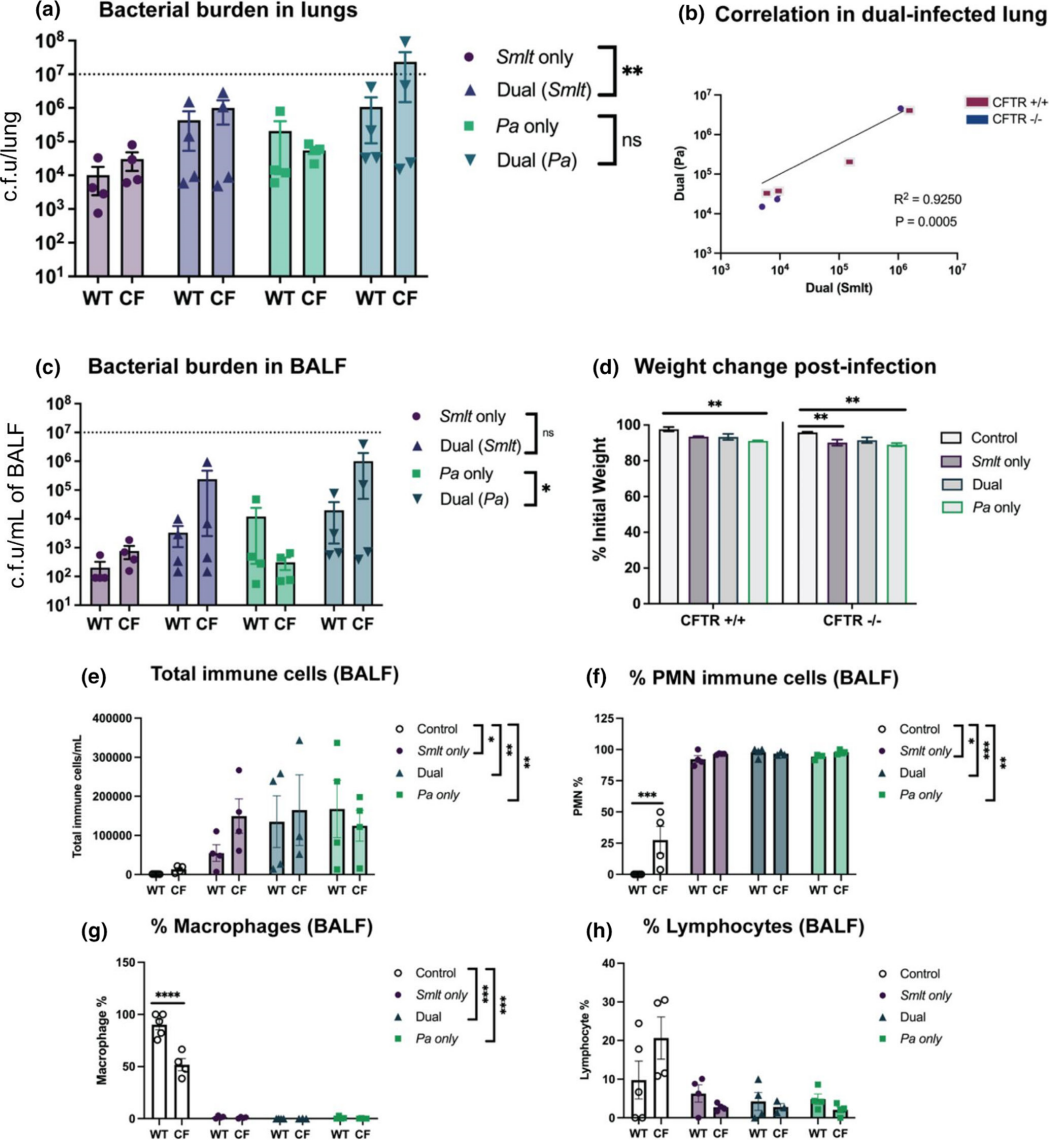
*
Stenotrophomonas maltophilia* and *Pseudomonas aeruginosa* dual-species respiratory infections in BALB/c Cftr^tm1UNC^ mice. BALB/c Cftr^tm1UNC^ mice and WT littermates were intratracheally infected with ~10^8^ c.f.u. ml^−1^ of *S. maltophilia *, *P. aeruginosa *, or both, and lung were assessed 24 h post-infection. (a) Bacterial burden in the lung homogenate (*N*=4). (b) The correlation between *S. maltophilia* and *P. aeruginosa* counts from lung homogenate and BALF (*N*=8). Linear regression with two-tailed Spearman correlation. (c) Bacterial burden in the BALF (*N*=4). (d) Weight loss was measured as percent initial weight. Dotted line indicates inoculum (*N*=4). Two-way ANOVA with Sidak’s multiple comparison test. Mean±sem, **P<0.01. BALF was collected to differentially count immune cells, including (e) total immune cells, (f) PMN percentage, (g) macrophages percentage and (h) lymphocyte percentage (*N*=4). Comparisons between CF mice and WT littermates were evaluated by two-way ANOVA with Sidak’s multiple comparison test. Statistical comparisons between bacterial infection groups were performed using Kruskal–Wallis with Dunn’s multiple comparison test. Mean±sem, *P<0.05, **P<0.01, ***P<0.001.

In our previous work, we have shown that *Stenotrophomonas maltophilia* and *Pseudomonas aeruginosa* exist within polymicrobial biofilms on the lung mucosal surface, where polymicrobial infection results in reduced bacterial clearance of one or both organisms and increased disease consequences compared to infection with either organism [13]. Following previously established methods, we evaluated this phenotype in a BALB/c Cftr^tm1UNC^ mouse model. CFTR mutant mice and their WT littermates were intratracheally infected with *S. maltophilia* and *P. aeruginosa* alone, or in a concurrent polymicrobial infection (Fig S1B). Mice were euthanized for bacterial quantification and assessment of host response 24 h post-infection. As expected, polymicrobial infection resulted in increases in bacterial burden of *S. maltophilia* compared to single-species infections (**P<0.01), with no statistically significant differences observed in bacterial counts between WT and CF mice within each group (Fig. 3a). *S. maltophilia* and *P. aeruginosa* showed a linear relationship between bacterial counts for each species in the coinfected mouse groups (R^2^=0.9250) (Fig. 3b). In addition to counts from lung homogenate, bacterial burden in the BALF also showed increased counts from dual-infected animals compared to a single-species infection (*P<0.05). There was no difference in bacterial counts from the BALF between WT and CF mice (Fig. 3c). Percent initial weight post-infection showed that bacterial infection impacts morbidity compared to uninfected controls, with *P. aeruginosa* single-species infection resulting in the most significant weight loss for both WT and CF mice (**P<0.01) (Fig. 3d). *S. maltophilia* and *P. aeruginosa* infection did not result in increased mortality at 24 h post-infection (Table S2). The immune cell infiltrates counted from BALF showed that all infection groups resulted in a significant increase in total immune cells compared to uninfected controls; however, there was no difference in total immune cell counts between WT and CF mice (Fig. 3e). The composition of these total immune cells showed increased PMN following bacterial infection for all infection groups compared to uninfected controls, with polymicrobial infection contributing to significant influx (***P<0.001). Percentages of PMN were significantly higher in uninfected CF mice compared to uninfected WT littermates (***P<0.001); however, there was no difference in WT and CF mice in any other infection groups (Fig. 3f). In a proportional manner, macrophage and lymphocyte composition of total immune cells was higher for uninfected control mice compared to other infection groups. Macrophage percentage was significantly higher in WT uninfected controls compared to CF uninfected controls (****P<0.0001). For all other infection groups, there was no difference between WT and CF mice (Fig. 3g-h). These findings establish that *S. maltophilia* and *P. aeruginosa* colonization is significantly correlated in BALB/c Cftr^tm1UNC^ mice and that consequences of dual-species infection indicate a cooperative relationship.

**Fig. 4.**
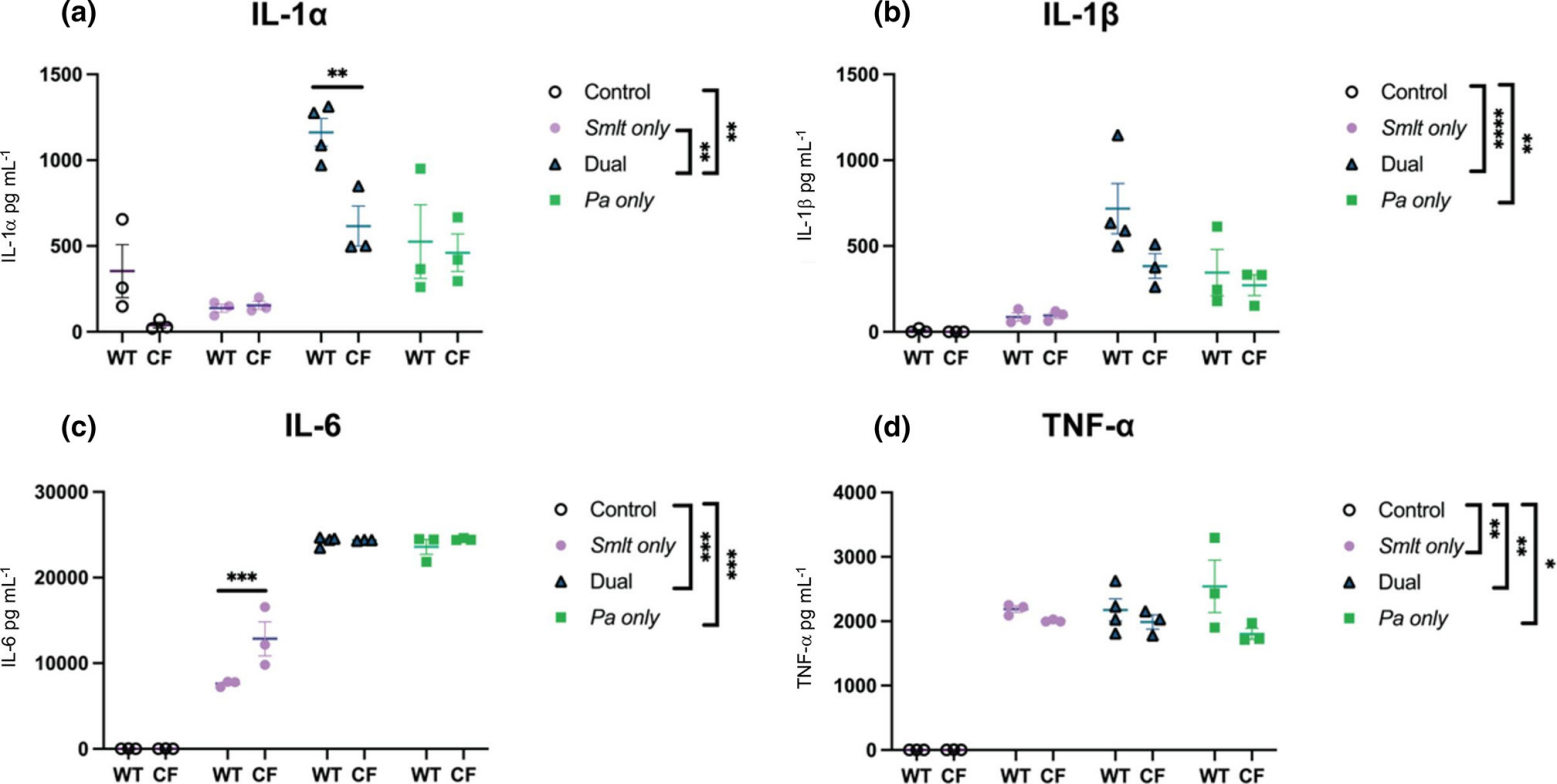
Inflammatory response following *Stenotrophomonas maltophilia* and *Pseudomonas aeruginosa* dual-species infection in BALB/c mice.Cytokine/chemokine analyses of BALF supernatant from infected or mock-infected BALB/c Cftr^tm1UNC^ mice and wild-type littermates following *S. maltophilia* and *P. aeruginosa* infection. Luminex multiplex assay measured (a) IL-1α, (b) IL-1β, (c) IL-6 and (d) TNF-α. (*N*=3–4). Comparisons between CF mice and their WT littermates were evaluated by two-way ANOVA with Sidak’s multiple comparison test. Mean±sem, *P<0.05, **P<0.01. Comparisons between bacterial infection groups were evaluated by Kruskal–Wallis with Dunn’s multiple comparison test. Mean±sem, *P<0.05, **P<0.01, ****P<0.0001.

Previously, immunological consequences of *S. maltophilia* and *P. aeruginosa* dual-species infections result in higher levels of IL-1α, IL-1β, IL-6, MIP-2 and MCP-1 in dual-infected mice compared to a *S. maltophilia* single-species infection, with no difference in TNF-α between infection groups [13]. To test these immunological trends in the BALB/c Cftr^tm1UNC^ mouse model, we measured inflammatory markers in the BALF by multiplex assay for IL-1α, IL-1β**,** IL-6 and TNF-α. This assay showed similar results, in which IL-1α levels were significantly increased in dual-infected mice compared to *S. maltophilia* only infection (**P<0.01), and IL-1β levels showed a trending increasing in dual-infected mice compared to *S. maltophilia* infection. There was no difference between WT and CF groups, with an exception being increased IL-1α levels in dual-infected WT mice (**P<0.01) (Fig. 4a-b). IL-6 measurements increased following dual-infection as compared to *S. maltophilia* single-species infection, with a significant increase following dual-infection between CF and WT groups (***P<0.001) (Fig. 4c). TNF-α levels were not different between infection groups and were not different between WT and CF mice within each infection group, with an exception being an increase in WT mice following *P. aeruginosa* single-species infection (*P<0.05).

### *S. maltophilia* and *P. aeruginosa* lead to lung pathology in a C57BL/6J- Cftr^tm1UncTg^(FABPhCFTR)1Jaw/J mouse model

**Fig. 5.**
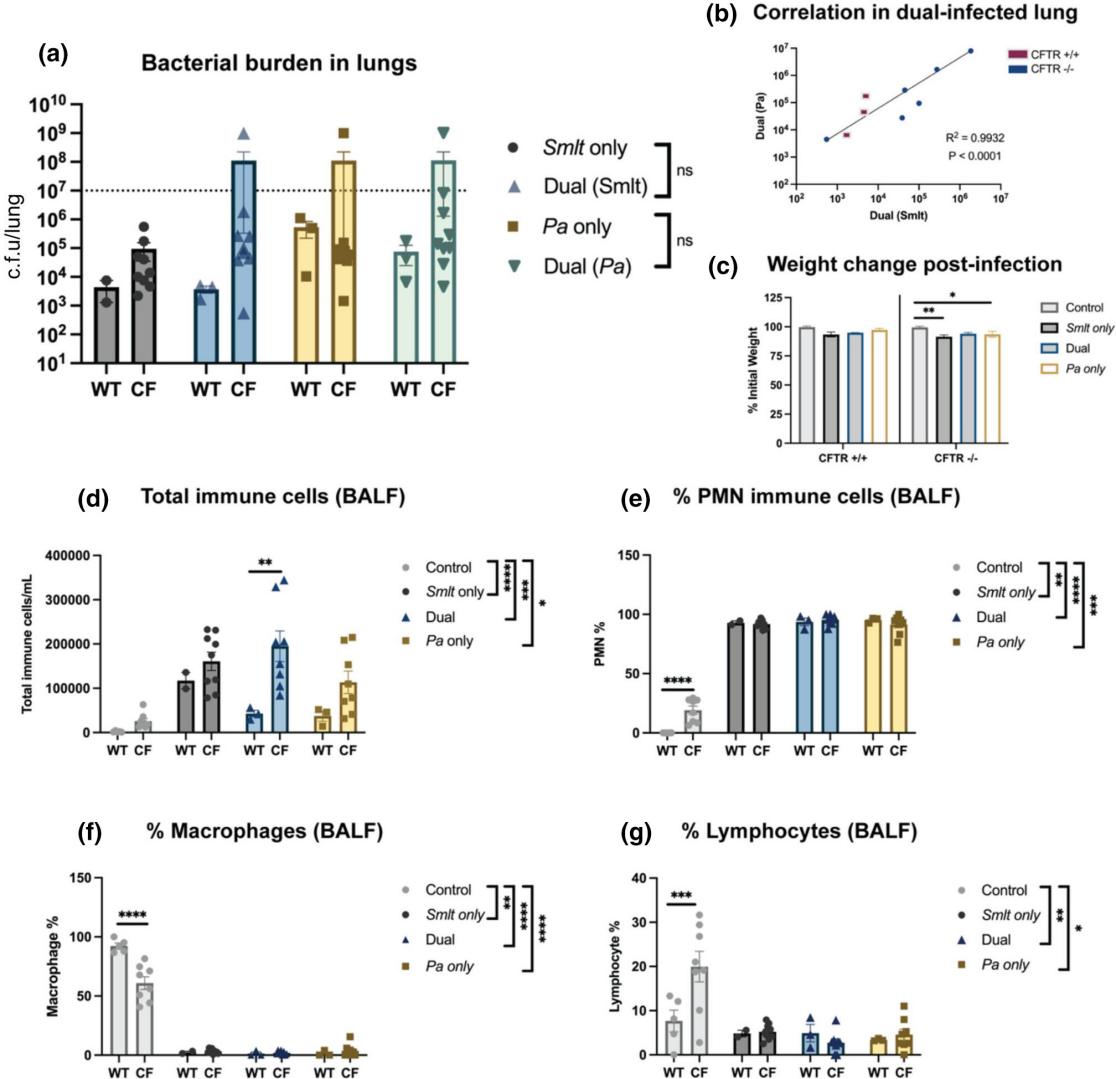
*
Stenotrophomonas maltophilia* and *Pseudomonas aeruginosa* polymicrobial respiratory infections in C57BL/6J-Cftr^tm1UncTg^(FABPhCFTR)1Jaw/J mice. C57BL/6J-Cftr^tm1UncTg^(FABPhCFTR)1Jaw/J mice and WT littermates were intratracheally infected with ~10^8^ c.f.u. ml^−1^ of *S. maltophilia *, *P. aeruginosa *, or both, and lung were assessed 24 h post-infection. (a) Bacterial burden in the lung homogenate was enumerated by viable colony counting (*N*=2–9). (b) Correlation between *S. maltophilia* and *P. aeruginosa* in the lung (*N*=9). Linear regression with two-tailed Spearman correlation. (c) Weight loss was measured as percent initial weight at 24 h post-infection. Two-way ANOVA with Sidak’s multiple comparison test. Mean±sem, *P<0.05, **P<0.01. BALF was collected to differentially count immune cells in the lung lumen, including (d) total immune cells, (e) the percentage of total cells that are PMN, (f) the percentage of total cells that are macrophages and (g) the percentage of total cells that are lymphocytes. Comparisons between CF mice and their WT littermates were evaluated by two-way ANOVA with Sidak’s multiple comparison test. Statistical analyses of infection data were performed using Kruskal−Wallis with Dunn’s multiple comparison test (*N*=2–9). Mean±sem, *P<0.05, **P<0.01, ***P<0.001, ****P<0.0001.

While a cooperative relationship between *S. maltophilia* and *P. aeruginosa* was not altered by immunological response in the BALB/c Cftr^tm1UNC^ CF mice, we wanted to test this polymicrobial relationship, as well as evaluate metrics of infection consequences, in the C57BL/6J- Cftr^tm1UncTg^(FABPhCFTR)1Jaw/J mice, which are thought to display more CF-like pathology than BALB/c Cftr^tm1UNC^ mice [14]. This experiment allows for a direct comparison of experimental infections performed in independently derived CF mutant mice with separate host lineages. Following previously established methods, we intratracheally infected C57BL/6J- Cftr^tm1UncTg^(FABPhCFTR)1Jaw/J mice and age matched WT littermates with *S. maltophilia* and *P. aeruginosa* alone, or in a concurrent, polymicrobial infection (Fig S1B). At 24 h post-infection, there were no significant difference in bacterial burden of *S. maltophilia* and *P. aeruginosa* in lung homogenates following single-species or dual-species infections. There was also no difference in bacterial counts between WT and CF mice within each group (Fig. 5a). Although *S. maltophilia* and *P. aeruginosa* did not appear to be synergistically increasing counts from polymicrobial infection compared to single-species infection, there was still a linear relationship between bacterial counts for each species (R^2^=0.9932) (Fig. 5b). In this study, bacterial infection leads to no more weight loss than uninfected controls for WT mice; however, CF mice lost more weight following both *S. maltophilia* (**P<0.01) and *P. aeruginosa* (*P<0.05) infection compared to uninfected controls (Fig. 5c). Both polymicrobial infection and *P. aeruginosa* single-species infection caused significant mortality in CF mice (11.1 % for each group) (Table S3). In BALF, all infection groups resulted in a significant increase in total immune cell infiltration compared to uninfected controls and there were similar trends in total immune cell counts between WT and CF mice, with an exception being an increased influx in CF mice following polymicrobial infection (**P<0.01) (Fig. 5d). The percentage of PMN were increased following bacterial infection for all groups compared to uninfected controls, with polymicrobial infection contributing to significant influx (****P<0.0001). Percentages of PMN were significantly higher in uninfected CF mice compared to uninfected WT littermates (****P<0.0001), however, there was no difference in WT and CF mice from any other infection groups (Fig. 5e). Proportionally, macrophage and lymphocyte composition of total immune cells were higher for uninfected control mice compared to other infection groups. Macrophage percentage was significantly higher in WT uninfected controls compared to CF controls (****P<0.0001) and lymphocyte percentage was significantly higher in CF controls compared to WT controls (***P<0.001). For all other infection groups, there was no difference in macrophage or lymphocyte percentages in BALF between WT and CF mice (Fig. 5f-g). Overall, these findings suggests that *S. maltophilia* and *P. aeruginosa* correlate in a C57BL/6J- Cftr^tm1UncTg^(FABPhCFTR)1Jaw/J mice, but that bacterial counts and weight loss were not significantly different between infection groups.

**Fig. 6.**
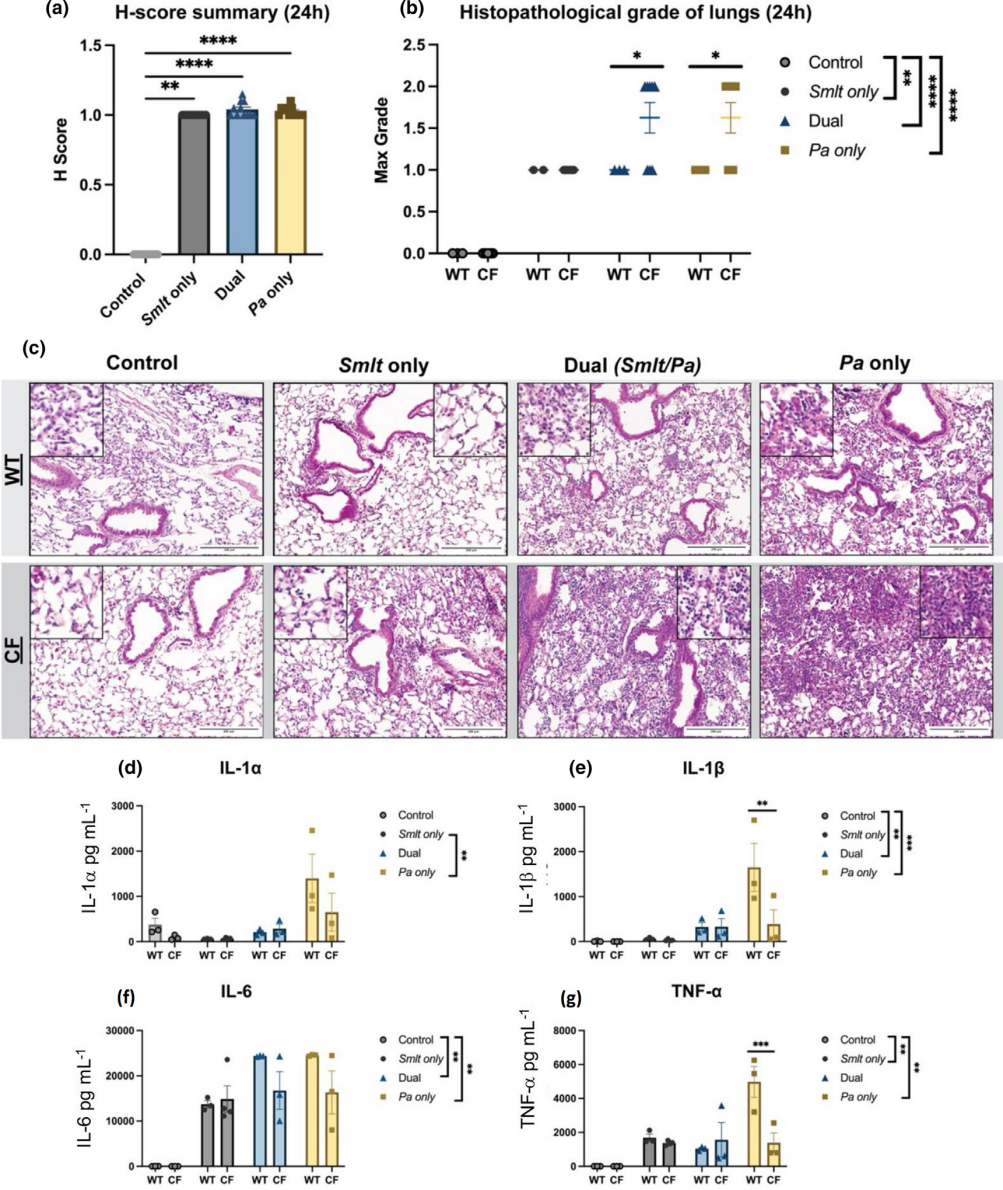
Histopathologic analyses and inflammatory responses in lungs from C57BL/6J-Cftr^tm1UncTg^(FABPhCFTR)1Jaw/J mice following infection with *Stenotrophomonas maltophilia* and *Pseudomonas aeruginosa *. Lung sections from C57BL/6J-Cftr^tm1UncTg^(FABPhCFTR)1Jaw/J mice and WT littermates were evaluated for tissue damage following *S. maltophilia* and *P. aeruginosa* dual-species infection. (a) H-score reflects a cumulative tissue grade (*N*=11). Kruskal–Wallis with Dunn’s multiple comparison test. Mean±sem, **P<0.01, ****P<0.0001. (b) Maximum grade scored per animal (*N*=2–9). Comparisons between CF mice and their WT littermates were evaluated by two-way ANOVA with Sidak’s multiple comparison test. Mean±sem, *P<0.05. Comparisons between bacterial infection groups were evaluated by Kruskal–Wallis with Dunn’s multiple comparison test. Mean±sem, **P<0.01, ****P<0.0001. Pathological grading by L.N. Representative images of (c) H and E-stained lung sections comparing CF mice and their WT littermates after bacterial infection. Rare macrophage presence in the alveoli of uninfected CF mice is not indicative of infection. Scale bar=200 µm. Cytokine/chemokine analyses of BALF supernatant from infected or mock-infected C57BL/6J-Cftr^tm1UncTg^(FABPhCFTR)1Jaw/J mice and WT littermates following *S. maltophilia* and *P. aeruginosa* infection. Luminex multiplex assay measured (d) IL-1α, (e) IL-1β, (f) IL-6 and (g) TNF-α (*N*=3–4). Comparisons between CF mice and their WT littermates were evaluated by two-way ANOVA with Sidak’s multiple comparison test. Mean±sem, **P<0.01, ***P<0.001. Comparisons between bacterial infection groups were evaluated by Kruskal–Wallis with Dunn’s multiple comparison test. Mean±sem, **P<0.01, ***P<0.001.

In previous studies, *S. maltophilia* and *P. aeruginosa* polymicrobial infection resulted in more damage to the lungs than *S. maltophilia* single-species infection [4]. Lung pathology of the C57BL/6J- Cftr^tm1UncTg^(FABPhCFTR)1Jaw/J CF mice is thought to be more inflamed and, therefore, more representative of CF disease [5, 6]. To test how dual-infection may influence lung pathology, we assessed the lungs of *S. maltophilia* and *P. aeruginosa* infected C57BL/6J- Cftr^tm1UncTg^(FABPhCFTR)1Jaw/J mice and their non-CF littermates. H and E-stained lung sections were evaluated and graded by a pathologist for lung damage based on alveolar, perivascular and peribronchial inflammation, pleuritis, and tissue necrosis on a scale of 0–3. Following *S. maltophilia* and *P. aeruginosa* infection, each infection group resulted in significant airway tissue damage from bacterial infection compared to uninfected control, with both dual-infection and *P. aeruginosa* only infection leading to severe lung tissue damage (****P<0.0001) (Fig. 6a). The maximum grade following infection shows similar findings, with polymicrobial infection and single-species *P. aeruginosa* infection resulting in the most severe damage compared to uninfected controls (****P<0.0001). When comparing WT and CF infection groups, CF mice had higher composite scores for tissue damage following dual-species and *P. aeruginosa* single-species infection (*P<0.05) (Fig. 6b). Representative images of these lung sections show the severity of tissue damage following polymicrobial and *P. aeruginosa* infections characterized by neutrophil influx and alveolar tissue necrosis (Fig. 6c). These findings indicate that cooperativity, or increased dual-species infection consequences, are diminished in the Cftr^tm1UncTg^(FABPhCFTR)1Jaw/J CF mouse model and the development of lung injury in response to infection may be more advanced.

To assess the immunological response of C57BL/6J- Cftr^tm1UncTg^(FABPhCFTR)1Jaw/J mice to *S. maltophilia* and *P. aeruginosa* polymicrobial infection, we measured inflammatory markers in the BALF by multiplex assay for IL-1α, IL-1β**,** IL-6 and TNF-α. Following bacterial challenge, IL-1α levels were significantly higher in *P. aeruginosa* infected mice compared to *S. maltophilia* single-species infection, but there was no difference between WT and CF groups (**P<0.01) (Fig. 6d). IL-1β measurements showed the most significant readings from dual-species infection compared to uninfected controls (***P<0.001). There was no difference between WT and CF groups, with an exception being WT mice showing increased IL-1β following *P. aeruginosa* single-species infection (**P<0.01) (Fig. 6e). IL-6 measurements showed an increase following polymicrobial infection and *P. aeruginosa* single-species infection compared to uninfected controls, but there was no significant difference between CF and WT groups (**P<0.01) (Fig. 6f). TNF-α levels were elevated in *S. maltophilia* single-species animals and *P. aeruginosa* single-species animals compared to uninfected controls (**P<0.001). TNF-α was also significantly higher in WT mice following *P. aeruginosa* single-species infection compared to CF mice (***P<0.001). There was no difference between WT and CF mice for other infection groups (Fig. 6g). Taken together, these results support the conclusion that the C57BL/6J- Cftr^tm1UncTg^(FABPhCFTR)1Jaw/J CF mouse model presents subtle differences in histopathological findings and inflammatory cytokine measurements between CF mice and their WT littermates. Despite these significant but subtle differences in host response, bacterial colonization during polymicrobial infections were comparable between CF and WT mice.

## Discussion

For people with CF, impaired CFTR function alters the ionic environment of the airways, a process that is thought to change the compartmental pH, reducing antimicrobial defenses, and promoting hyperinflammation [17–19]. It is well established that mucus in the CF airways, along with impaired function of host defensins and other antimicrobial factors normally found in airway surface liquid (ASL), leave patients susceptible to respiratory infection with pathogenic bacteria [17, 20]. Early-stage, acute infections require aggressive treatment, which influences the ecological shift in microbial community members over time towards those that recalcitrant to treatment [5]. As patients become persistently colonized, they experience chronic inflammation, which causes host tissue damage, stimulates mucus hypersecretion and impairs mucociliary escalator function, which in turn promotes further microbial persistence [21–23].

CF-associated infections have been extensively studied for many years, but one limiting factor has been the development and widespread availability of animal models that faithfully replicate the pathophysiology and susceptibility of patients with CF [24–27]. While it is now clear that CF mutant mice do not reliably develop spontaneous lung pathology typically observed in humans with CF disease, in some situations increased chronic inflammation and phagocytic influx into the airway in CF mice provide a model that is more susceptible to bacterial pathogens [28–33]. There remains much to be learnt about the strengths and limitations of these mice for pathogenesis studies, particularly with regard to the polymicrobial infections that are typical in the clinical course of CF disease.

The first CF mouse models of disease were generated soon after discovery of CFTR function. Of these original models, Cftr^tm1UNC^ was one of the first described, created by a traditional route of replacement of *CFTR* gene, which results in complete knockout with no normal CFTR function [32, 34]. Cftr^tm1UNC^ mice have been utilized for colonization and pathogenesis studies; however much remains to be learnt regarding polymicrobial CF infections in these mice [30, 33, 35].

Genetic background differences in BALB/c and C57BL/6 mice are known to lead to divergent immunological responses; BALB/c mice predominantly exhibit Th2 centred cellular immune responses whereas C57BL/6 mice have more T_h_1 dominant responses [7, 8]. Our initial infection studies focused on BALB/c Cftr^tm1UN^ mice. As previously reported for infection studies with BALB/c mice, bacterial challenge with nontypeable *H. influenzae* (NTHi) and *P. aeruginosa* resulted in reduction of *P. aeruginosa* bacterial burden, with reduced inflammatory response and airway tissue damage (Fig. 1 and Fig. 2). Bacterial challenge with *S. maltophilia* and *P. aeruginosa* resulted in cooperative infection with more significant inflammatory consequences following dual infection (Figs 3–5). These experiments are consistent with our published work seen in non-CF BALB/c mice [12, 13].

C57BL/6J Cftr^m1UNC^/Cftr^m1UNC^ mice were shown to have reduced mucociliary transport and evidence of fibrosis typical of that observed in patients with CF [30, 36]. One complicating issue was the development of gastrointestinal disease symptoms in these mice, which greatly impacted their survival [36]; this was later addressed by development of bitransgenic mice, expressing rat fatty acid binding protein 2 and an intestinal promoter for expression of human CFTR, creating a gut corrected model with increased survival while maintaining lung disease [14]. We used Cftr^tm1Unc^-Tg(FABPCFTR)1Jaw/J to evaluate lung pathology in a CF mouse as a consequence of acute bacterial infection.

Previous infection studies in Cftr^tm1Unc^-Tg(FABPCFTR)1Jaw/J and their non-CF littermates showed few differences in severity of lung disease following *P. aeruginosa* infection, but highlighted the lung functional decline associated with ageing [37]. We assessed the initial immune response aimed at bacterial clearance before lung function is thought to be impaired. Our work shows that *S. maltophilia* colonizes the lungs of Cftr^tm1Unc^-Tg(FABPCFTR)1Jaw/J at 24 h post-infection (Fig. 5A). We found that although polymicrobial infection with *S. maltophilia* and *P. aeruginosa* showed correlation in the lungs, bacterial burden and immune cell influx from polymicrobial infection did not differ significantly from that of single-species infection, indicating reduced cooperativity in this model (Fig. 5). As lung pathology representing CF disease in mouse models is uncertain, we tested the histopathological implications of *S. maltophilia* and *P. aeruginosa* infection in Cftr^tm1Unc^-Tg(FABPCFTR)1Jaw/J. Here, we note that Cftr^tm1Unc^-Tg(FABPCFTR)1Jaw/J mice showed slightly more severe pathological scores than their WT littermates, a finding represented by noticeable inflammation in lung tissue (Fig. S6). *S. maltophilia* and *P. aeruginosa* infection in Cftr^tm1Unc^-Tg(FABPCFTR)1Jaw/J resulted in increased influx of inflammatory cytokines, with notable increases in IL-1β and TNF-α in Cftr^tm1Unc^-Tg(FABPCFTR)1Jaw/J knockout mice following *P. aeruginosa* infection (Fig. 6). Significant influxes of these markers may indicate the magnitude of acute infection response that diverges amongst murine backgrounds.

This work is unique in evaluating the utility of several CF mouse models for study of polymicrobial respiratory infections, however, there are several limitations to consider while interpreting these results. Herein, all infections were introduced to CF mice with Cftr^tm1UNC^ mutations, only testing the interplay of one genetic mutation background in bacterial inter-species interactions out of the many CF mouse models. Infection studies assessing NTHi/*Pa* dual-species infection were only performed in the BALB/c background, a factor that should be evaluated in future work assessing the role of T-cell composition on the innate immune priming response. Additionally, other animal models of CF disease may replicate more CF-like respiratory anomalies, including the CF rats, ferrets and pigs, all of which harbour submucosal glands that CF mice do not [24]. As lung microbial communities are rich in inter-species relationships, moving forward these polymicrobial infection studies will incorporate other examples of microbial interactions, such as inter-species or inter-kingdom competition, or complex infections with more than two pathogens.

In summary, this work explores the applications of CF mice to polymicrobial interactions among relevant CF pathogens. We carried out polymicrobial bacterial infections in BALB/c Cftr^tm1UNC^ and Cftr^tm1UncTg^(FABPhCFTR)1Jaw/J mice, evaluating early infection response and bacterial clearance. As we have observed previously, NTHi infection caused innate immune priming that can reduce both colonization and pathology associated with subsequent *P. aeruginosa* infection, including bacterial burden, tissue damage and inflammatory response to *P. aeruginosa* [12]. Polymicrobial infections in the BALB/c Cftr^tm1UNC^ also showed increased bacterial load and increased infectious consequences, indicating significant cooperativity between *S. maltophilia* and *P. aeruginosa* populations *in vivo*. With these two examples of polymicrobial infections in BALB/c Cftr^tm1UNC^, this model exhibits very few differences in infectious response between CF mice and their WT littermates, highlighting the limitations of its application to infection studies. *S. maltophilia* and *P. aeruginosa* dual-infections in a Cftr^tm1UncTg^(FABPhCFTR)1Jaw/J show significant correlation, but not promotion between these two organisms. However, both lung pathology and inflammatory response to *P. aeruginosa* infection of Cftr^tm1UncTg^(FABPhCFTR)1Jaw/J were increased compared to their non-CF littermates. Overall, our findings suggest that these CF models are equally reliable for the study of polymicrobial infections compared to non-CF mice, but that the Cftr^tm1UncTg^(FABPhCFTR)1Jaw/J mouse model may provide a more severe pathological response to acute respiratory infection.

## Supplementary Data

Supplementary material 1
