# Supplementary material for: Acute polymicrobial airway infections: analysis in cystic fibrosis mice
Source: Microbiology (Reading). 2023 Jan 25;169(1):001290. doi: 10.1099/mic.0.001290 (PMC9993112; doi:10.1099/mic.0.001290)
Supplement: Supplementary material 1 [file mic-169-1290-s001.pdf]

Table S1: Total mortality* rates of NTHi/*Pa* infected BALB/c mice

	Infection Group	Total (Deaths)	Percent Mortality
WT	Control	4 (0)	0.0%
	NTHi only (~10^8)	8 (2)	25.0%
	NTHi/*Pa* Dual (~10^8/~10^7)	9 (0)	0.0%
	Pa only (~10^7)	9 (1)	11.1%
CF	Control	4 (0)	0.0%
	NTHi only (~10^8)	7 (1)	14.3%
	NTHi/*Pa* Dual (~10^8/~10^7)	8 (2)	25.0%
	Pa only (~10^7)	9 (4)	44.4%

Table S2: Total mortality* rates of *Smlt/Pa* infected BALB/c mice

	Infection Group	Total (Deaths)	Percent Mortality
WT	Control	3 (0)	0.0%
	Smlt only (~10^7)	4 (0)	0.0%
	Smlt/Pa Dual (~10^7/~10^7)	4 (0)	0.0%
	Pa only (~10^7)	4 (0)	0.0%
CF	Control	4 (0)	0.0%
	Smlt only (~10^7)	4 (0)	0.0%
	Smlt/Pa Dual (~10^7/~10^7)	4 (0)	0.0%
	Pa only (~10^7)	4 (0)	0.0%

Table S3: Total mortality* rates of *Smlt/Pa* infected C57BL/6 mice

	Infection Group	Total (Deaths)	Percent Mortality
WT	Control	5 (0)	0.0%
	Smlt only (~10^7)	2 (0)	0.0%
	Smlt/Pa Dual (~10^7/~10^7)	3 (0)	0.0%
	Pa only (~10^7)	3 (0)	0.0%
CF	Control	9 (0)	0.0%
	Smlt only (~10^7)	9 (0)	0.0%
	Smlt/Pa Dual (~10^7/~10^7)	9 (1)	11.1%
	Pa only (~10^7)	9 (1)	11.1%

*Mortality is defined as death prior to the end of the study

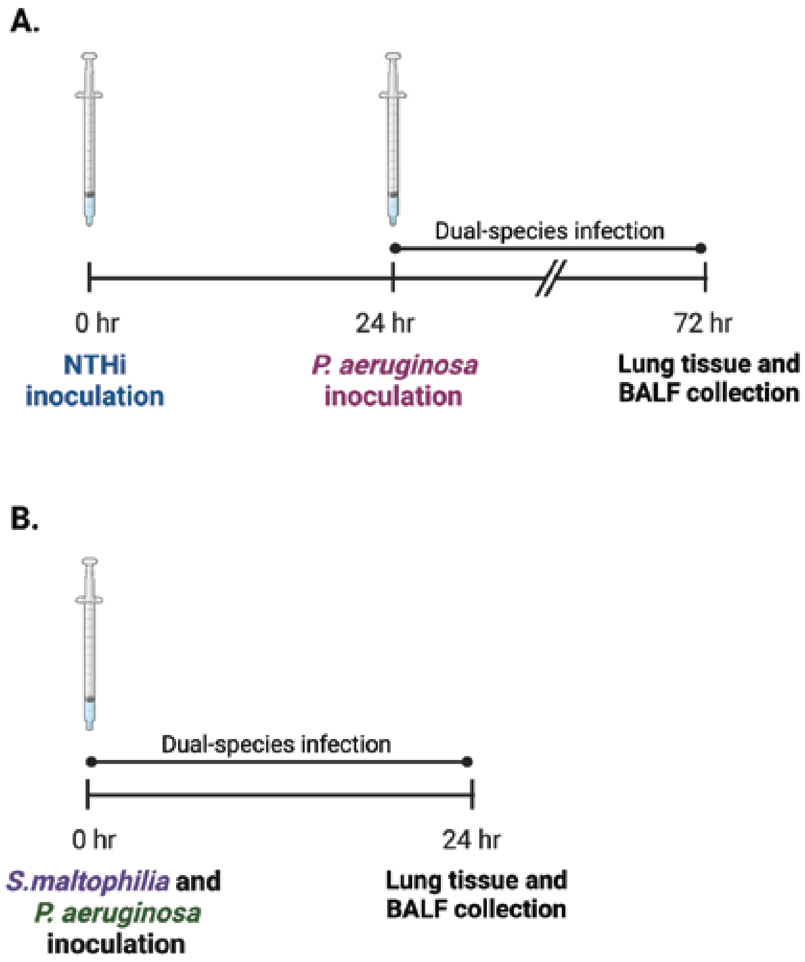

Figure S1: Timelines of polymicrobial infection.
Mice were sequentially infected with A) nontypeable *Haemophilus influenzae* (NTHi) 24 hours prior to inoculation with *P. aeruginosa*. At 72 hours post- initial infection (48 hours post- dual infection), the lung tissue and BALF were collected. Mice were concurrently infected with B) *S. maltophilia* and *P. aeruginosa* and the lung tissue and BALF were collected at 24 hours post-infection.
